# Peer Review of “Modeling Years of Life Lost Due to COVID-19, Socioeconomic Status, and Nonpharmaceutical Interventions: Development of a Prediction Model”

**DOI:** 10.2196/38420

**Published:** 2022-04-12

**Keywords:** COVID-19, pandemic, socioeconomic status, mortality, nonpharmaceutical interventions, prediction model, low-income status, life expectancy, public health, income groups

*This is a peer-review report submitted for the paper “Modeling Years of Life Lost Due to COVID-19, Socioeconomic Status, and Nonpharmaceutical Interventions: Development of a Prediction Model.”*

## Review Round 1

This paper [1] develops a model that compares the years of life lost (YLL) due to COVID-19 and the potential YLL due to the socioeconomic consequences of its containment. The results highlight the importance of socioeconomic status (SES) in evaluating the effect of nonpharmaceutical interventions (NPIs) during COVID-19. However, the methods, especially the empirical sample characteristics from which the life table is derived, are not clear.

### Specific Comments

#### Major Comments

1. Needs to describe more about the data, study design, and study sample in more detail

2. Needs to discuss how the missing data was handled

3. It is important to consider a theoretical framework that can guide the selection of NPIs, indicators of SES, and the equivalent socioeconomic damages (on page 11). Right now, it is more arbitrary than scientific based.

4. The Discussion also needs to consider other factors (eg, pre-existing conditions, neighborhood resources, or occupation types). These are important social determinants of health factors

#### Minor Comments

1. The tables need to be adjusted in terms of the decimal points and more informative legends to guide readers.

## Abbreviations

NPI: nonpharmaceutical intervention
SES: socioeconomic status
YLL: years of life lost
